# *O*-Linked *N*-Acetylglucosamine Transiently Elevates in HeLa Cells during Mitosis

**DOI:** 10.3390/molecules23061275

**Published:** 2018-05-26

**Authors:** Viktória Fisi, Emese Kátai, József Orbán, Silvia Dossena, Attila Miseta, Tamás Nagy

**Affiliations:** 1 Department of Laboratory Medicine, Medical School, University of Pécs, Pécs H7624, Hungary; fisi.viktoria@gmail.com (V.F.); mesekatai@gmail.com (E.K.); miseta.attila@pte.hu (A.M.); 2 Department of Biophysics, Medical School, University of Pécs, Pécs H7624, Hungary; josesan77@gmail.com; 3 Institute of Pharmacology and Toxicology, Paracelsus Medical University, Salzburg 5020, Austria; silvia.dossena@pmu.ac.at; 4 János Szentágothai Research Centre, University of Pécs, Pécs H7624, Hungary

**Keywords:** cell cycle, cell synchronization, mitosis, *O*-GlcNAc, post-translational modifications

## Abstract

*O*-linked *N*-acetylglucosamine (*O*-GlcNAc) is a dynamic post-translational modification of serine and threonine residues on nuclear and cytoplasmic proteins. *O*-GlcNAc modification influences many cellular mechanisms, including carbohydrate metabolism, signal transduction and protein degradation. Multiple studies also showed that cell cycle might be modulated by *O*-GlcNAc. Although the role of *O*-GlcNAc in the regulation of some cell cycle processes such as mitotic spindle organization or histone phosphorylation is well established, the general behaviour of *O*-GlcNAc regulation during cell cycle is still controversial. In this study, we analysed the dynamic changes of overall *O*-GlcNAc levels in HeLa cells using double thymidine block. *O*-GlcNAc levels in G_1_, S, G_2_ and M phase were measured. We observed that *O*-GlcNAc levels are significantly increased during mitosis in comparison to the other cell cycle phases. However, this change could only be detected when mitotic cells were enriched by harvesting round shaped cells from the G_2_/M fraction of the synchronized cells. Our data verify that *O*-GlcNAc is elevated during mitosis, but also emphasize that *O*-GlcNAc levels can significantly change in a short period of time. Thus, selection and collection of cells at specific cell-cycle checkpoints is a challenging, but necessary requirement for *O*-GlcNAc studies.

## 1. Introduction

Checkpoints between cell cycle phases (G_0_ to G_1_, G_1_ to S, S to M and M to G_1_) refer to a more or less well-defined transition from one regulatory mechanism to the next one; certain proteins become deactivated while others are activated. Although it has the shortest duration, M phase can be further divided into sub-phases characterized by distinct signaling processes and distinct events. Each step in the cell cycle is influenced by the previous steps and is affected by hundreds of regulatory elements. Alterations to the progression of cell cycle and its regulatory mechanisms due to either intrinsic (mutations) or extrinsic (environmental changes, chemicals, etc.) causes may have serious consequences; most notably, the pathological changes of the cell cycle could lead to uncontrolled proliferation, but disturbance in the cell cycle regulation has been also associated with Alzheimer’s disease, diabetes or autoimmune diseases [1,2,3].

Protein phosphorylation and its dynamic changes during the various stages of the cell cycle were extensively studied; the central elements of the cell cycle regulation: cyclins, cyclin dependent kinases and their upstream regulators and downstream targets are known to activate or inhibit signaling elements by phosphorylation [4]. Around cyclins and cyclin dependent kinases, dozens of other kinases and phosphorylases are tightly organized both spatially and temporally. Many of their functions are well understood, and the periodic activity (and inactivity) of these proteins during the cell cycle is well established. However, hundreds of other cyclin-independent regulatory proteins may be also involved in the regulation of the cell cycle [5]. Besides phosphorylation, ubiquitination is also a well-known process involved in cell cycle regulation. Ubiquitination of a particular protein is a sign that the protein is selected for degradation. During various stages of the cell cycle, various sets of signaling proteins could be deemed to degradation [6]. Besides degradation, ubiquitination also regulates protein trafficking, scaffolding, DNA damage repair, and chromatin topology [7]. It is also worth noting that ubiquitination and phosphorylation may strongly interact; e.g., phosphorylation can target proteins for ubiquitination [8].

Although phosphorylation and ubiquitination are the most studied post-translational modifications participating in the signal transduction, dozens of other types of post-translational modifications (e.g., SUMOylation or Poly ADP-Ribosylation) have been also recognized to influence cell signaling [9,10]. Among these, scientific interest increased in recent years toward the so-called *O*-linked β-*N*-acetylglucosamine, or *O*-GlcNAc protein modification. *O*-GlcNAc seems to be an important regulator/modulator of many intracellular functions, including cell cycle [2]. *O*-GlcNAc is the enzymatic addition of a single *N*-acetyl-glucosamine molecule to the OH residues of serine or threonine. This mechanism is reversible, and in contrast to other types of glycosylation, it takes place in the cytoplasm and nucleus instead of the ER. The number of known *O*-GlcNAc sites and the proteins carrying those sites are rapidly increasing thanks to recent advances in mass spectrometry techniques—over 1000 *O*-GlcNAc proteins have been identified so far [11]. *O*-GlcNAc can interact with other post-translational modifications, most notably it can compete with phosphorylation for the same sites [12]. Interestingly, *O*-GlcNAcylation may also protect against polyubiquitination and increase protein stability [13,14,15,16,17].

A unique aspect of *O*-GlcNAc modification is that it is influenced by the metabolic state of the cell. Its substrate, UDP-GlcNAc, is produced by the hexosamine biosynthesis pathway (HBP), which is sustained by approx. 1–3% of the glucose entering the cell. It was recognized early that one of the functions regulated by *O*-GlcNAc is nutrient sensing [18]. Since then, many other cellular functions were demonstrated to be influenced by *O*-GlcNAc, including Ca^2+^ signaling, epigenetics, stress adaptation and notably: cell cycle [19,20]. Several studies reported that manipulating *O*-GlcNAc regulation can disrupt the normal progress of the cell cycle [21,22,23]. *O*-GlcNAc has been shown to be required for histone phosphorylation [24,25], mitotic spindle organization [26] or for the expression of genes associated with DNA replication and cell cycle progression such as *c-myc* [27]. *O*-GlcNAc is also thought to be part of the histone code [28]; others dispute these results [29]. The participation of *O*-GlcNAc in the regulation of cell cycle is undeniable; however, its role is far from being completely understood. *O*-GlcNAc levels were found to either increase [22,23,30,31,32] or decrease [24,33] during mitosis. This controversy could be—at least in part—explained by the reversible nature of *O*-GlcNAc modification. Some events of the cell cycle, especially during mitosis, occur in a few minutes, thus making it difficult to capture cells representative for a particular cell cycle phase.

The aim of the present study was to analyse the possible changes of *O*-GlcNAc levels in HeLa cells during various cell cycle stages. With the combination of synchronization of the cells by double thymidine block and fractionated cell collection, we have detected increased *O*-GlcNAc levels during mitosis. Thus, our findings support the idea that *O*-GlcNAc is elevated in mitosis and also demonstrate the highly dynamic nature of *O*-GlcNAc regulation.

## 2. Results

### 2.1. Synchronization of HeLa Cells by Double Thymidine Block

**Figure 1:**
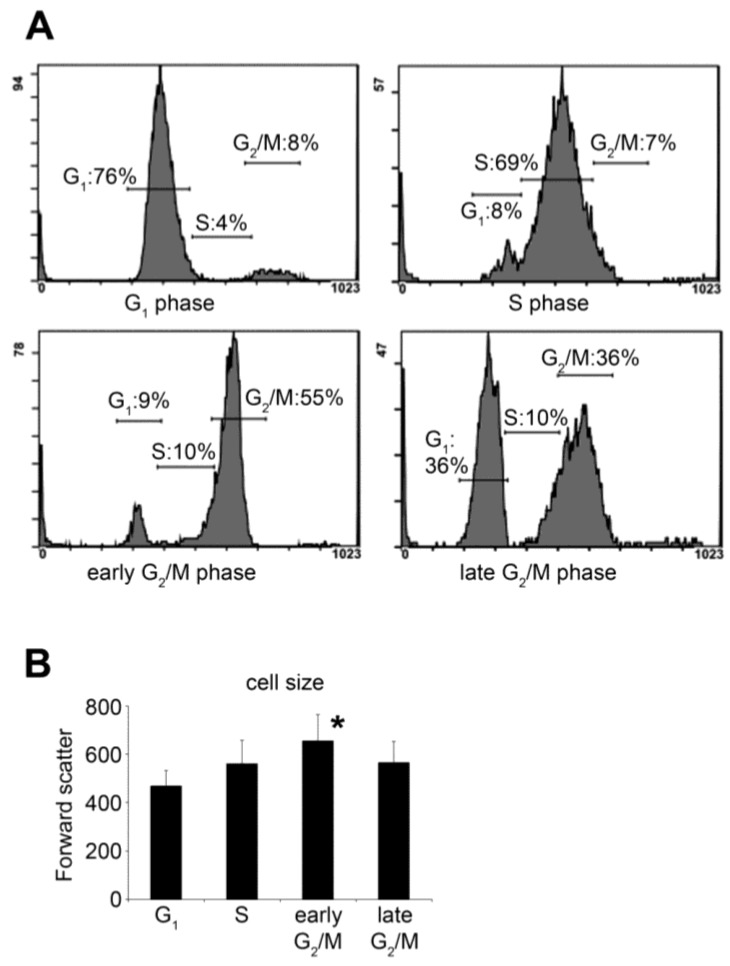
Characterization of cell cycle dynamic after double thymidine block. (**A**) HeLa cells were synchronized with double thymidine treatment and then allowed to recover from the blockage. Cell cycle distribution was monitored every 4 h by flow cytometry and propidium-iodide staining after fixation of the cells. Cell cycle distributions are shown as histogram plots of the FL3 fluorescence channel. G_1_, S, early G_2_/M and late G_2_/M phases were captured 0, 4, 8 and 12 h after the release of the block, respectively. Please note that the terms “early” and “late” refer to the average status of the total population, not the current position of individual cells; (**B**) average cell size was analysed by measuring the forward scatter (FS) values of live cells using flow cytometry. Cells were collected 0, 4, 8 and 12 h after the release of the block to obtain representative data for G_1_, S, early G_2_/M and late G_2_/M phases. FS is proportional to the size of the cells, and shows that the cell size increases during the cell cycle progression and reaches a peak in the early G_2_/M phase. Data are shown as means ± SD from at least three independent experiments, * *p* < 0.05.

We have synchronized HeLa cells by a double thymidine block, which synchronizes cells at the G_1_-S border [34,35]. At the end of the treatment, the large majority of the cells were synchronized at the G_1_ phase. According to the DNA content of the cells determined by propidium-iodide, four hours after the release of the block the cells progressed into S phase and eight hours after most of the cells reached the G_2_ phase. After 12 h, approximately half of the synchronized cells underwent mitosis (Figure 1). The duration of each cell cycle phases in synchronized cell cultures is longer than the transit time of an individual cell in that particular phase. The transit time for the total population (*T_total_*) can be calculated by the following equation:

*T_d_* + *T_Phase_* = *T_total_*,

where *T_d_* is the delay between the first and the last cell entering a given cell cycle phase, and $T_{Phase}$ is the average time a single cell spends in that phase [36]. In line, we use the term ‘early G_2_/M phase’ to describe the time period when most cells are in G_2_ phase but did not undergo mitosis yet, and ‘late G_2_/M phase’ to describe the time period a significant number of cells already underwent mitosis, while the rest of the cells are about to enter mitosis.

### 2.2. Selective Collection of Mitotic Cells Resulted in Detection of Distinct Changes in O-GlcNAc Pattern

**Figure 2:**
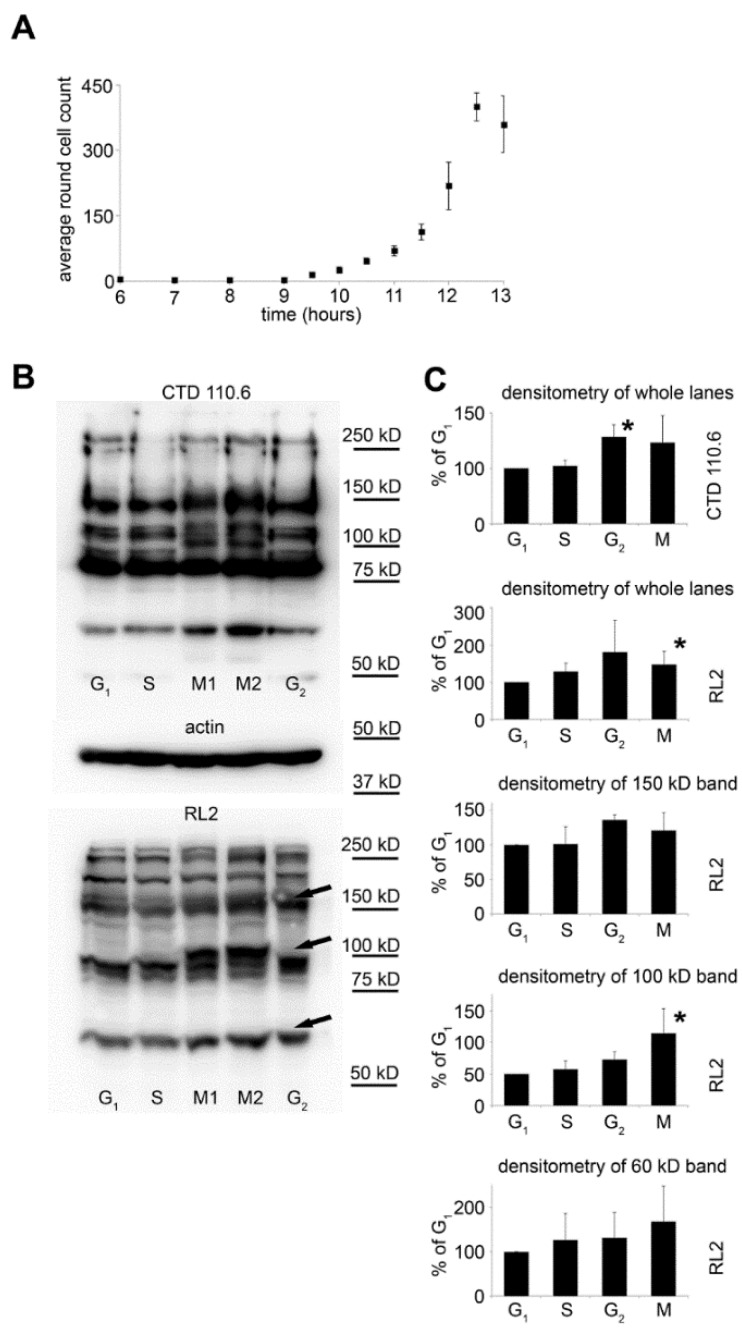
Overall protein *O*-GlcNAc levels during various cell cycle phases. (**A**) HeLa cells were synchronized and allowed to recover from the thymidine blockage. The number of mitotic cells at the indicated times was assessed as the number of round shaped cells per field of view using bright-field microscopy. Data are shown as means ± SD from at least 3 independent experiments; (**B**) representative Western blot analysis using RL2 or CTD110.6 anti-*O*-GlcNAc antibody and anti-actin antibody on total protein extracts isolated from synchronized cells. Cells were collected at specific cell cycle phases as described in the Materials and Methods. For the mitotic cells, we pooled the first six fractions (M1) and the last three fractions (M2) prior to lysis; (**C**) densitometric analysis of CTD110.6 staining of whole lanes in the four main cell cycle phases (top panel) and densitometric analysis of RL2 staining of whole lanes and three individual bands (at ~150, 100 and 60 kD, as indicated by arrows in (**B**) in the four main cell cycle phases. Data from M1 and M2 fractions were combined for statistical analysis. Data are shown as means ± SD, * *p* < 0.05 vs. G_1_.

Although in our synchronized cultures up to 70% of the cells were in the same phase, the individual mitotic events are spread over several hours. To have a better estimation of the number of cells actually undergoing mitosis during shorter time frames (20–25 min.), we have counted the round shaped cells at regular intervals in synchronized HeLa cultures. Figure 2A shows that the number of round shaped cells started to rise 9 h after synchronization, reaching peak counts between 12–13 h post-synchronization.

Based on this result, we modified our sample collection protocol for Western blotting to collect mitotic cells in ~25 min. fractions from 9 to 13 h after synchronization by vigorously shaking the cell culture flasks to detach these cells from the surface. The first six fractions (M1) and the last three fractions (M2) were pooled together. Moreover, in this set of experiments, all samples were lysed directly in Laemmli sample buffer; consequently, the lysate represented the protein content of the whole cell. Figure 2B shows overall *O*-GlcNAc levels detected by Western blotting. For comparison, G_1_ phase, S phase, and G_2_ phase cells were also harvested and analysed. Overall, *O*-GlcNAc levels were analysed by RL2 and CTD110.6 antibodies. Both antibodies detected altered *O*-GlcNAc patterns in the mitotic and G_2_ fractions, compared to other cell cycle phases. Most notably, a strong protein band at ~100 kD appeared in the mitotic cells (Figure 2B,C).

### 2.3. Synchronization Alone Is Not Sufficient to Detect Changes in Overall O-GlcNAc Levels during G_2_/M–G_1_ Transition

**Figure 3:**
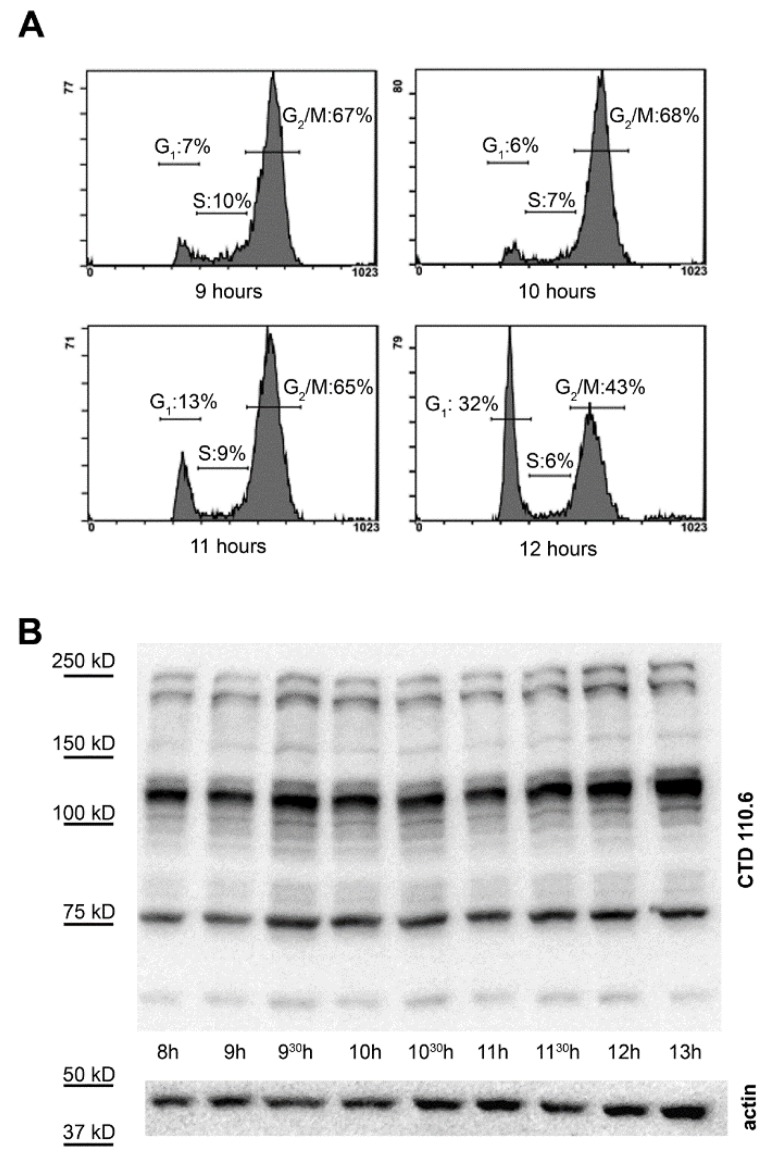
Overall protein *O*-GlcNAc levels during G_2_/M phase. (**A**) HeLa cells were synchronized and the DNA content was monitored prior and during the G_2_/M–G_1_ transition (9, 10, 11 and 12 h after the release of the thymidine block) by flow cytometry and propidium-iodide staining; (**B**) representative Western blot using CTD110.6 anti-*O*-GlcNAc and anti-actin antibodies on total protein extracts isolated from synchronized cells at the indicated times after thymidine block release.

We measured the cell cycle distribution prior and during the time most cells progressed from G_2_/M phase to G_1_ phase. For this, HeLa cells were synchronized by double thymidine block and were allowed to progress in their cell cycle for 9, 10, 11 or 12 h. Our data showed that mitosis started between 10–11 h post synchronization and about half of the cells (6% G_1_ cells at 10 h vs. 32% G_1_ at 12 h and 68% G_2_/M at 10 h vs. 43% G_2_/M at 12 h) already passed mitosis 12 h post synchronization (Figure 3A).

Therefore, we collected several fractions of cells from synchronized cultures for protein isolation and *O*-GlcNAc detection in a time period overlapping the critical phase when most cells progressed through mitosis (8–13 h). *O*-GlcNAc levels were detected by Western blotting with CTD110.6 anti-*O*-GlcNAc antibody. As Figure 3B demonstrates, no extra band or any obvious trend or change over time could be recognized during the G_2_/M–G_1_ transition.

### 2.4. Nuclear Pore Proteins Are Associated with Variable O-GlcNAc Content during Cell Cycle

**Figure 4:**
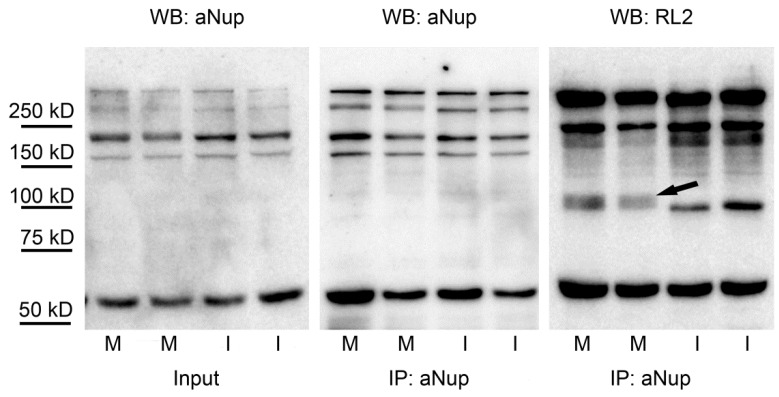
*O*-GlcNAc modification of nuclear pore proteins in mitotic and interphase cells. Mitotic and interphase cells were separated from asynchronous HeLa cells by mitotic shake-off. Nuclear pore proteins were enriched by immunoprecipitation with mouse monoclonal antibody MAb414 (aNup). Duplicates samples from mitotic (M) and interphase (I) cells were assessed by Western blot; detecting nuclear pore proteins with aNup antibody in crude cell extracts (input) and in immunoprecipitates (IP) and *O*-GlcNAc modified proteins with RL2 antibody in immunoprecipitates. The arrow indicates an extra *O*-GlcNAc band appearing at ~100 kD in the immunoprecipitates of the mitotic samples.

Nuclear pore proteins (Nups) are among the longest known, heavily *O*-GlcNAc modified proteins [37]. *O*-GlcNAc modification was shown to be necessary for the correct expression of nuclear pore proteins [38]. On the other hand, it may also change the structural property of the pores and interfere with nuclear transport and permeability [39]. Since very few data are available on the regulation of Nups by *O*-GlcNAc, we have enriched Nups using mouse monoclonal antibody MAb414 to assess *O*-GlcNAc levels in mitotic cells. Corresponding to previous data, we have found that Nups are abundantly *O*-GlcNAc modified (Figure 4). Moreover, at ~100 kD molecular weight in mitotic cells, we have found an *O*-GlcNAc staining pattern similar to that observed in crude cell extracts (Figure 2B). In addition, we have tested the anti-Nup immunoprecipitates for the presence of OGT and OGA. Unfortunately, we could not find any association of either OGT or OGA with Nup proteins in any of our samples (data not shown), although crude cell extracts contained both OGT and OGA (Figure S1).

### 2.5. Immunofluorescence Detection Shows Increased Level of O-GlcNAc in Mitotic HeLa Cells

**Figure 5:**
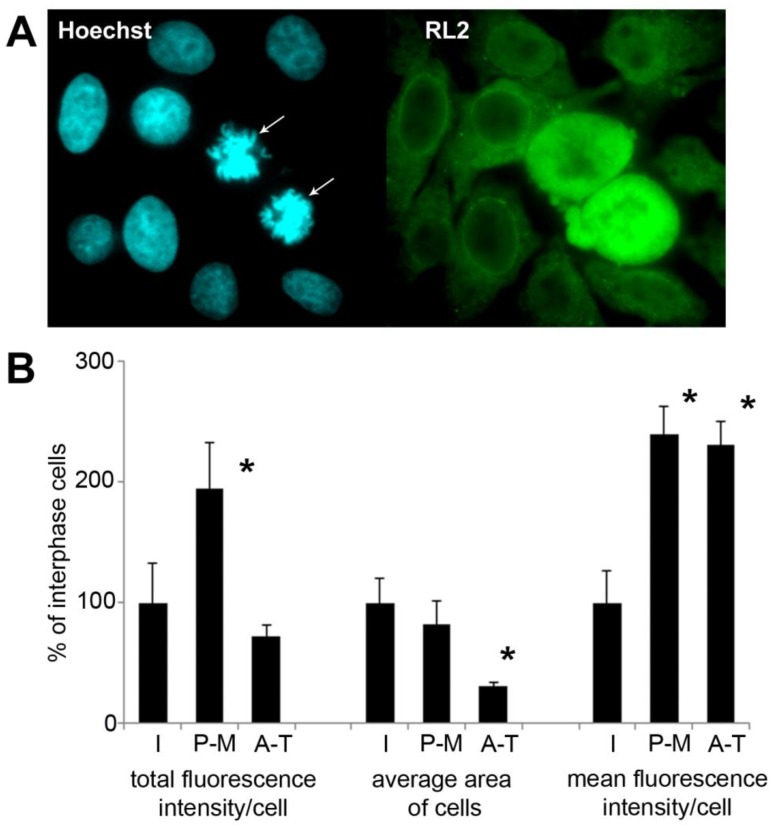
Immunofluorescence shows dynamic protein *O*-GlcNAc regulation during mitosis. Immunofluorescence labelling was performed on asynchronous HeLa cells grown and fixed on glass coverslips. (**A**) representative images captured by epi-fluorescence microscopy of cell stained with RL2 (green) and Hoechst (blue, nucleus) are shown. Two cells in mitosis (prometaphase) are indicated by arrows; (**B**) bar graph shows total fluorescence intensity per cell, area per cell and the average fluorescence intensity per cell (total fluorescence intensity divided by the area of the cell). Based on the morphology, the cells were categorized in three groups: interphase (I), prophase-metaphase (P-M) and anaphase-telophase (A-T). Data are expressed as percentage compared to the interphase cells in the same field of view. Each bar represents the average of 10 cells for P-M and A-T cells and 50 cells for interphase cells. Data are shown as means ± SD, * *p* < 0.05 vs. interphase.

To assess the amount of *O*-GlcNAc and its subcellular distribution at the level of individual cells, asynchronous HeLa cells grown on coverslips were formalin-fixed and labelled with anti-*O*-GlcNAc monoclonal antibody RL2. As shown in Figure 5A, cells during mitosis (confirmed by Hoechst nuclear counterstaining) showed elevated levels of *O*-GlcNAc. To quantify this, we measured fluorescence intensity levels from interphase and mitotic cells (Figure 5B). Mitotic cells were further divided into early (prophase-metaphase (P-M)) and late (anaphase-telophase (A-T)) mitotic cells. Mitotic cells demonstrated significantly higher fluorescence intensity than interphase cells. The total fluorescence of the cells late in the mitosis was significantly lower. This was expected since the size of the daughter cells is approx. 50% of the metaphase cells before the division. Average fluorescence intensity in A-T cells was similar to the level of P-M cells after normalizing fluorescence intensity by the size of the cells.

**Figure 6:**
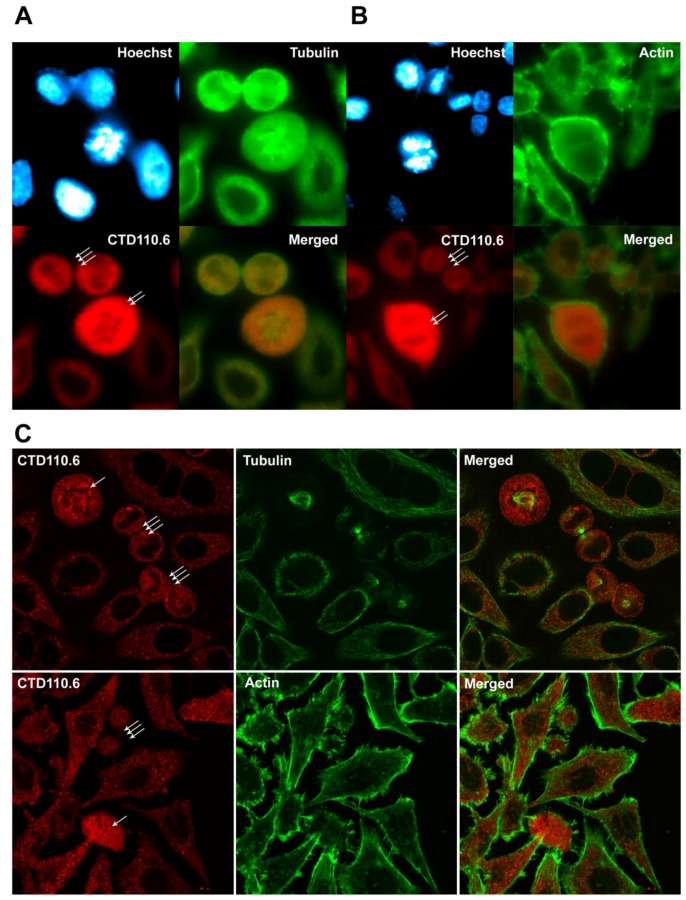
Protein *O*-GlcNAc distribution pattern compared to tubulin and actin staining. Immunofluorescence labelling was performed on asynchronous HeLa cells grown and fixed on glass coverslips. (**A**) representative epi-fluorescence images of cells stained with CTD110.6 (red), an anti-tubulin antibody (green) and Hoechst nuclear staining (blue), showing HeLa cells in various cell cycle phases; (**B**) representative images of asynchronous HeLa cells captured by epi-fluorescence microscopy in various cell cycle phases. Cells were stained with CTD110.6 (red), phalloidin-Alexa Fluor 488 (actin, green) and Hoechst (blue, nucleus); (**C**) confocal microscopy images showing asynchronous HeLa cells stained with CTD110.6 (red) and tubulin or actin (green) and corresponding merged images. Mitotic cells are indicated by arrows (prometaphase: single arrow, anaphase: double arrow, telophase: triple arrow).

We have also investigated the relationship between tubulin and actin cytoskeletal proteins and *O*-GlcNAc modification during mitosis. Tubulin and actin has been previously demonstrated to be part of the ever growing *O*-GlcNAc protein family [40,41]. Thus, we simultaneously labelled asynchronous HeLa cells with CTD110.6 and an anti-tubulin antibody (Figure 6A) or phalloidin-Alexa Fluor 488 for actin (Figure 6B). CTD110.6 staining showed similar results to RL2, and the strong fluorescence signal of the mitotic cells was clearly distinct from the signal of the interphase cells. Mitotic cells containing the mitotic spindles visualized by tubulin staining also demonstrated increased level of *O*-GlcNAc. Epi-fluorescence microscopy did not show any obvious morphologic similarity between *O*-GlcNAc and either tubulin or actin staining pattern, thus we also examined possible co-localization by confocal microscopy. Figure 6C shows representative confocal images of asynchronous HeLa cells, including interphase and mitotic cells. In both interphase and mitotic cells, *O*-GlcNAc staining was granular and relatively evenly distributed in the cytoplasm. In mitotic cells, CTD110.6 labelling seemed to be excluded from the region where the chromosomes were localised–similarly CTD110.6 was also excluded from the nuclear region of interphase cells (in contrast to RL2, CTD110.6 is a mouse IgM antibody, which cannot penetrate the nuclear envelope). On the other hand, tubulin and actin both showed fibrillar morphology and a very different distribution pattern than *O*-GlcNAc. Based on our results, a large-scale co-localization between *O*-GlcNAc and either tubulin or actin could be ruled out, but potential *O*-GlcNAc modification of a smaller subset of actin or tubulin unfortunately remains hidden in this experimental setup.

## 3. Discussion

In the present study, we analysed the dynamic changes of global *O*-GlcNAc levels in HeLa cells during various cell cycle stages following synchronization by double thymidine block. We report here that *O*-glycosylation pattern did not alter during the G_1_ and S phases, but a sudden elevation in M phase occurred. These changes remained hidden without the use of mitotic shake-off, which ensures a precise, almost pure mitotic cell collection [33]. According to our immunofluorescence results, this modification lasts only for a short period in the telophase of the mitosis, and, at the time of cytokinesis, the amount of *O*-glycosylated proteins already decreases.

There is increasing evidence representing *O*-GlcNAc modification as an important coordinator of the cell cycle. e.g., the level of *O*-GlcNAc-transferase (OGT) and *O*-GlcNAcase (OGA) the enzymes responsible for *O*-GlcNAc addition and removal were shown to be significantly fluctuate during various cell cycle transitions, correlating with changes of *O*-GlcNAc levels [19,32]. Thus, nutritional substrate availability for *O*-GlcNAc modification could be a pre-requisite for cell cycle entry. A decrease of *O*-glycosylation caused by reduced OGT activity (either by gene deletion or by substrate depletion) in mouse embryonic fibroblasts was associated with cell growth defects like delayed growth, increased expression of the cyclin dependent kinase inhibitor p27 and cell death [2,21,42,43].

Information about *O*-glycosylation of individual proteins during the cell cycle is also available. Important players of mitogen signaling PI3K and MAPK together with cyclin D1, a key regulator of G_1_ phase have been reported to be disabled by blocking OGT after serum-stimulation [44,45]. All four core nucleosomal histones (H2A, H2B, H3, and H4) are *O*-GlcNAc modified in a cell cycle-dependent manner [28,46]. G_2_/M transition highly depends on cyclin B1 expression, which is decreased by either OGT or OGA inhibition showing that both enzymes are needed for proper cell cycle progression [47]. The other regulator of M phase entry, the cyclin B regulated CDK1 (cyclin dependent kinase) is also influenced by *O*-GlcNAc [48]. At the time of mitosis, OGT localizes to the mitotic spindle and later during cytokinesis to the midbody [49]. It has also been reported that OGT together with OGA plays a role in the regulation of the stability of the midbody through acting in a complex with the mitotic kinase Aurora B and protein phosphatase 1 [49]. In the anaphase, CDH1, one of the co-activators of anaphase promoting complex/cyclosome is shown to be *O*-GlcNAcylated, and this modification stimulates its activity [50]. Several transcription factors like myc, p53, NFkB and many others playing important role in cell division and tumorigenesis are also influenced by *O*-GlcNAc [47].

The changes of global *O*-GlcNAc level during the cell cycle are controversial. Several reports showed that an increase in *O*-glycosylation and OGT expression occurs in G_2_ phase and these events are necessary to G_2_/M transition [23,31,32,41,47,51]. It was also shown that OGT-inhibition impairs G_2_/M transition in Xenopus laevis oocytes [31]. According to Yang et al., *O*-GlcNAc starts to elevate already in the late S phase and reaches a peak in the M phase of MEF and HEK293 cells [32]. However, other authors did not find any change or even detected a decrease in global *O*-glycosylation and OGT expression during M phase [21,33,52]. Differences between these findings regarding *O*-GlcNAc changes during mitosis may be explained by the rapid and reversible nature of *O*-GlcNAc modification but can also arise from the different synchronization techniques and timing of sample collection. To study these rapid changes, various techniques were developed to synchronize cells such as serum starvation, double thymidine block or cell cycle progression arrest with chemical agents such as nocodazole or paclitaxel [36,53]. However, synchronization could be ineffective (the individual cell cycle events are “spread out” in time, thus only a small fraction of the cells are truly representative of an assumed phase) or the drugs used to block the cell cycle progress are toxic [48,52,53,54]. These agents, while effectively arresting cells, could also have a number of side-effects that might influence *O*-GlcNAc regulation. These agents, while effectively arresting cells, could also have a number of side-effects that might influence *O*-GlcNAc regulation. Most relevant for our present findings is the study of Sakabe et al., in which they found decreased *O*-GlcNAc levels in M phase compared to G_1_ phase [33]. In their experiment, they synchronized HeLa cells with nocodazole, mitotic shake-off and then replated the cells (for 1, 3 or 5 h) before final harvesting. In contrast, in our present study, mitotic cells were collected without the presence of any cell cycle arresting drug. A second difference could be that we collected mitotic cells in short (20–25 min.) fractions, thus ensuring higher homogeneity. Moreover, we used strong lysis conditions to include all cellular proteins in our samples while Sakabe et al. discarded NP-40 insoluble material by pelleting.

Previous reports suggest that *O*-GlcNAc influences cytoskeletal proteins during mitosis. In M phase cells, OGT was shown to co-localize with the mitotic spindle and vimentin is reported to be influenced by *O*-GlcNAc cycling enzymes [21,49]. Other studies have also found strong evidence that cytoskeletal elements are regulated by *O*-GlcNAc modification [26,48,55,56]. In our experiments, we did not find any detectable co-localization between *O*-GlcNAc and actin or tubulin. Based on the intracellular localization and morphology of the abundant *O*-GlcNAc during mitosis, the elevation of *O*-GlcNAc is probably not directly connected to cytoskeletal re-arrangement, but it is more likely the result of a distinct mechanism in the mitotic cells. However, the present study was limited by the abundance of *O*-GlcNAc, which hinders the study of co-localization with other proteins by confocal microscopy. Slight morphological changes and interactions with cytoskeletal proteins may have remained hidden. Thus, we do not exclude the possibility that key elements of the cytoskeletal system might be reversibly *O*-GlcNAcylated during mitosis. However, it seems improbable that the large increase of *O*-GlcNAc during mitosis would be caused solely by the re-arrangement of the cytoskeletal system. One of the limitations of our study could be that nuclear pore proteins (Nups) are strongly *O*-GlcNAc modified [37], which could skew the analysis of the immunofluorescently labelled interphase cells. However, it seems to be that Nups also persist during mitosis [57,58]. Our Western blot data, which agreed with the immunofluorescence analysis, was based on strong lysis conditions (Laemmli sample buffer), thus the lysate represented the total protein content, including nuclear pore proteins in both interphase and mitotic samples as well. Moreover, we have also analysed the level of *O*-GlcNAc modification on Nups by immunoprecipitation. While we have found both in mitotic and interphase cells abundantly *O*-GlcNAc modified Nups, distinct differences could be observed that corresponded to *O*-GlcNAc staining patterns found in whole cell lysates. This finding suggests that at least part of the changes we see during mitosis may be attributed to either Nups or other proteins that are strongly associated with Nups. The distinct *O*-GlcNAc band appearing in Nup immunoprecipitates at ~100 kD would be of particular interest to further analyze by more specific techniques such as mass spectrometry.

Although we are only starting to understand the impact of *O*-GlcNAc on the regulation of cell division, there is evidence that the cell cycle control and metabolism are tightly connected. In a modern lifestyle, an important risk factor for cancer is excessive food intake [59]. Since *O*-GlcNAc is an important signalling element in nutrient sensing, metabolic changes could impact cell cycle regulation through *O*-GlcNAc’s regulatory effect. Indeed, malignant cells show both an increased nutrient uptake and elevated *O*-GlcNAcylation [47]. It is also imperative that *O*-GlcNAc regulation in non-cancerous cells should be characterized in the future. A few studies using *Xenopus laevis* oocytes or embryonic fibroblasts showed an apparent increase in *O*-GlcNAc levels [30,32]; however, the data are far from complete and the difference between malignant and not malignant cells should be quantified. In fact, in the future, *O*-GlcNAcylation detection may become a valuable diagnostic and prognostic marker. Some authors have already proposed the measurement of OGT and OGA mRNA in urine as a diagnosis tool in bladder cancer [60]. Moreover, *O*-GlcNAc related proteins, e.g., OGT, OGA or *O*-GlcNAcylated cell cycle regulatory proteins, could represent promising targets in the therapy of malignant diseases [61].

## 4. Materials and Methods

### 4.1. Cell Line and Culture Conditions

HeLa cells were grown in a 1:1 mixture of EMEM and Ham’s F12 medium supplemented with 10 *v*/*v%* fetal bovine serum (FBS), 1 *v*/*v%* non-essential amino acids, penicillin (100 U/mL) and streptomycin (100 µg/mL). The cells were incubated at 37 °C, in 95% air-5 *v*/*v%* CO_2_ atmosphere in a humidified incubator. Subculturing was performed every 2–3 days and medium was refreshed 12–24 h prior to each experiment.

Synchronized cell cultures were created by double thymidine block [35,62]. Briefly, HeLa cells were grown in tissue culture flasks until ~40% confluency. In addition, 2 mM thymidine was added to the cell culture medium and the cells were incubated for 19 h at 37 °C. Next, the cells were incubated for 9 h in complete medium without thymidine. Finally, another 2 mM thymidine was added to the medium for 16 h. At the end of the process, the large majority of the cells were in G_1_ phase (Figure 1A).

For Western blot experiments, the cells were collected after synchronization as follows: G_1_ phase cells were collected by scraping immediately after the end of the double thymidine block treatment. S phase cells were collected by scraping 4 h after thymidine block release. Mitotic cells were collected in 20–25 min. fractions between 9–13 h post-synchronization by vigorously shaking the culture flask to detach round-shaped cells. G_2_ phase cells were collected by scraping the still attached cells after the last fraction of round-shaped cells were removed. Where indicated, mitotic cells were also isolated from asynchronous cell cultures by a similar fractionated shake-off method, while corresponding interphase cells were isolated from asynchronous cell cultures by scraping attached cells after a single vigorous shake-off removed all mitotic cells.

### 4.2. Flow Cytometry

Synchronized HeLa cells were detached from the cell culture vessels by incubation in phosphate-buffered saline (PBS) containing 0.25% trypsin and 0.5 mM EDTA for 3 min. at 37 °C. Resuspended cells were washed in complete medium to neutralize trypsin, next in PBS and then fixed in 1 mL ice-cold ethanol. The fixed cells were kept at 4 °C for at least 15 min, then washed with PBS 3X and resuspended in propidium-iodide (PI) solution (PBS, 0.1% Triton-X 100, 20 μg/mL PI, 0.2 mg/mL RNase A). After 30 min. incubation in the dark at room temperature, the fluorescence intensity of PI dye per cell was detected at 620 nm (FL3 channel) with a Cytomics FC 500 flow cytometer (Beckman Coulter, Fullerton, CA, USA). Gating and selection of regions (G_1_, S and G_2_/M phase) were performed on asynchronous, control cells and identical selections were utilized for all samples.

Cell size analysis was performed on live cells measuring the forward scatter (FS) values of synchronized cells. Briefly, HeLa cells were washed and resuspended in PBS at room temperature. FS was measured from each sample within 30 min with Cytomics FC 500 flow cytometer. Average FS values were obtained after collecting data from 10,000 events and gating out cell debris and events that were caused by clumped cells (<10% of the total events).

### 4.3. Cell Counting

Synchronized HeLa cells were allowed to enter the cell cycle after double thymidine block. At the indicated times (Figure 3A), the cells were observed with a Leica DM IL inverted microscope (Leica Microsystems, Wetzlar, Germany) and the average number of round-shaped cells per field of view were counted using a 10× objective from at least 5 separate regions of the flask. After each counting, round-shaped cells were discarded by vigorously shaking the flasks; consequently, only newly formed mitotic cells were included in the next counting.

### 4.4. Western Blot Analysis

HeLa cells were washed twice in ice-cold PBS buffer and harvested in RIPA buffer (10 mM Tris pH 7.2, 100 mM NaCl, 1 mM EDTA, 1 mM EGTA, 0.1 *w*/*v*% SDS, 1 *v*/*v%* Triton-X 100, 0.5 *w*/*v*% deoxycholate, 10 *v*/*v%* glycerol, protease inhibitor cocktail: 1 tablet/10 mL (Roche Applied Science, Penzberg, Germany), kept on ice for 30 min, and centrifuged for 10 min at 4 °C at 3000 rpm. From the supernatant, the total protein concentration was determined using a Bio-Rad DC Assay Kit (Bio-Rad, Hercules, CA, USA). Next, the samples were completed by the addition of 4X Laemmli sample buffer and boiled for 5 min. Where specified, the centrifugation step was omitted to obtain samples containing all proteins including RIPA-insoluble ones. For immunoprecipitated samples, we lysed the cells in IP buffer (25 mM Tris pH 7.2, 150 mM NaCl, 1 mM EDTA, 1 *v*/*v%* Triton-X 100, 5 *v*/*v%* glycerol, 1 tablet/10 mL protease inhibitor cocktail, 0,05 *w*/*v*% Na-azide and 100 µM *O*-(2-acetamido-2-deoxy-d-glucopyranosylidene)-amino-*N*-phenylcarbamate (PUGNAc)). Immunoprecipitation was done overnight at 4 °C with mouse monoclonal antibody MAb414 (Biolegend, San Diego, CA, USA, Cat. No.: 902902) followed by 3 h incubation with Protein A-Sepharose (Sigma-Aldrich, St. Louis, MO, USA, Cat. No.: P3391). Captured proteins were eluted by 0.1 M glycine, pH 2.8 and completed by the addition of 4X Laemmli sample buffer and boiled for 5 min.

Proteins were separated by 8 *w*/*v*% SDS-PAGE and transferred onto Polyvinylidene difluoride (PVDF) membranes (Millipore, Billerica, MA, USA). Blots were probed with the anti-*O*-GlcNAc antibody CTD110.6 (monoclonal mouse IgM (Sigma-Aldrich, Cat. No.: O7764, 1:2000) in 1 *w*/*v*% casein blocking buffer followed by horseradish peroxidase (HRP) conjugated goat anti-mouse IgM (Thermo Fisher Scientific, Waltham, MA, USA, 1:5000). Blots were also probed with mouse (monoclonal) anti-*O*-GlcNAc antibody RL2 (Thermo Fisher Scientific, Cat. No.: MA1-072, 1:1000), rabbit polyclonal anti–actin antibody (Sigma-Aldrich, Cat. No.: A2103, 1:1500) and mouse monoclonal antibody MAb414 (1:1000) according to the manufacturers’ protocol followed by their respective HRP conjugated secondary antibodies (1:2500). The blots were developed using Femto chemiluminescent substrate (Thermo Fisher Scientific) and the signal was visualized by Kodak Image Station 2000R (Eastman Kodak Company, Rochester, NY, USA). Kodak 1D (ver3.6.1, Eastman Kodak Company, NewHaven, CT, USA) and ImageJ (ver 1.51j, National Institutes of Health, Bethesda, Maryland, USA) analysis software were used to quantify the intensity of bands.

### 4.5. Immunofluorescence Microscopy

HeLa cells were grown on coverslips until ~50% confluency. Next, cells were washed twice in ice-cold PBS and fixed in 10 *v*/*v%* PBS-buffered formaldehyde for 30 min at room temperature. To avoid formaldehyde autofluorescence, the coverslips were quenched with 50 mM ammonium chloride for 10 min. The cells were permeabilized with 0.25 *v*/*v%* Triton-X 100 for 10 min. Nonspecific sites were blocked with 5% bovine serum albumin (Sigma-Aldrich) in PBS for 30 min. and then the coverslips were incubated at room temperature with the primary antibody (or antibodies) for 2 h in 5 *w*/*v*% BSA/PBS. The primary antibodies and their dilutions used were the following: CTD110.6 (1:200), RL2 (1:100), and anti-α-tubulin (Sigma-Aldrich, Cat. No.: T8203, 1:100). After rinsing 3 times with PBS, the samples were incubated with the secondary antibody for 1 h in dark. For visualizing actin, phalloidin-Alexa Fluor 488 conjugate (Thermo Fisher Scientific, Cat. No.: A12379, 1:20) was used. Nuclei were counterstained with Hoechst dye at a concentration of 0.24µg/mL for 15 min at room temperature. Finally, coverslips were mounted with Vectashield (Vector Laboratories, Burlingame, CA, USA) mounting medium. Image acquisition was performed with a Zeiss Axiovert 35 (Carl Zeiss Microscopy GmbH, Jena, Germany) inverted fluorescence microscope equipped with CellD software (ver2.6, Olympus Soft Imaging Solutions GmbH, Münster, Germany). Confocal images were captured using a Zeiss LSM 710 confocal scanning microscope (Carl Zeiss AG, Oberkochen, Germany) equipped with ZEN software (ver2.3, Carl Zeiss AG, Oberkochen, Germany) and a 63X objective. Alexa Fluor 488 and Texas Red fluorescence channels were used for image acquisition (ex.: 488 nm and 594 nm, em: 518 nm and 624 nm, respectively).

### 4.6. Data Analysis

Data are presented as means ± standard deviations (SD) throughout. Comparisons were performed using Student’s *t*-test. Statistically significant differences between groups were defined as *p*-values < 0.05 and are indicated in the legends of figures.

## Supplementary Materials

The following are available online at http://www.mdpi.com/1420-3049/23/6/1275/s1, Figure S1: OGT and OGA expression in mitotic and interphase cells.
