# Supplementary material for: O-Linked N-Acetylglucosamine Transiently Elevates in HeLa Cells during Mitosis
Source: Molecules. 2018 May 26;23(6):1275. doi: 10.3390/molecules23061275 (PMC6100377; doi:10.3390/molecules23061275)
Supplement: Supplementary file 1 [file molecules-23-01275-s001.pdf]

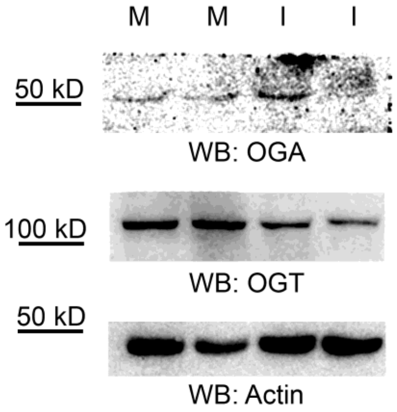

Supplementary Figure 1. OGT and OGA expression in mitotic and interphase cells. Mitotic and interphase cells were separated from asynchronous HeLa cells by mitotic shake-off. Samples from mitotic (M) and interphase (I) cells were assessed by Western blot; detecting *O*-GlcNAc transferase (Abcam, Cambridge, UK, Cat. No.: 184198) and *O*-GlcNAcase (Abnova, Taipei City, Taiwan, Cat. No.: H00010724-M02) in crude cell extracts (input) with specific antibodies. Anti-actin antibody was used as loading control.
